# Supplementary material for: Enhancing the Deposition Rate and Uniformity in 3D Gold Microelectrode Arrays via Ultrasonic-Enhanced Template-Assisted Electrodeposition
Source: Sensors (Basel). 2024 Feb 15;24(4):1251. doi: 10.3390/s24041251 (PMC10893058; doi:10.3390/s24041251)

[Thu Dec 28 11:59:14 2023] L-Edit V2016.2 File Name: WAFER-3DEL-4 Final Mask-For paper.tdb Cell: ChipFinalLayout_40Etch:WAFER-3DEL-4 Final Mask-For paper Scale: 10.5137

M2>Passivation_Layout_SiOx
Grid Layer
M1>MEA_Layout_Cr/Au
M3>Template_Layout_KMPR
M1>Scribe_Line_Cr/Au

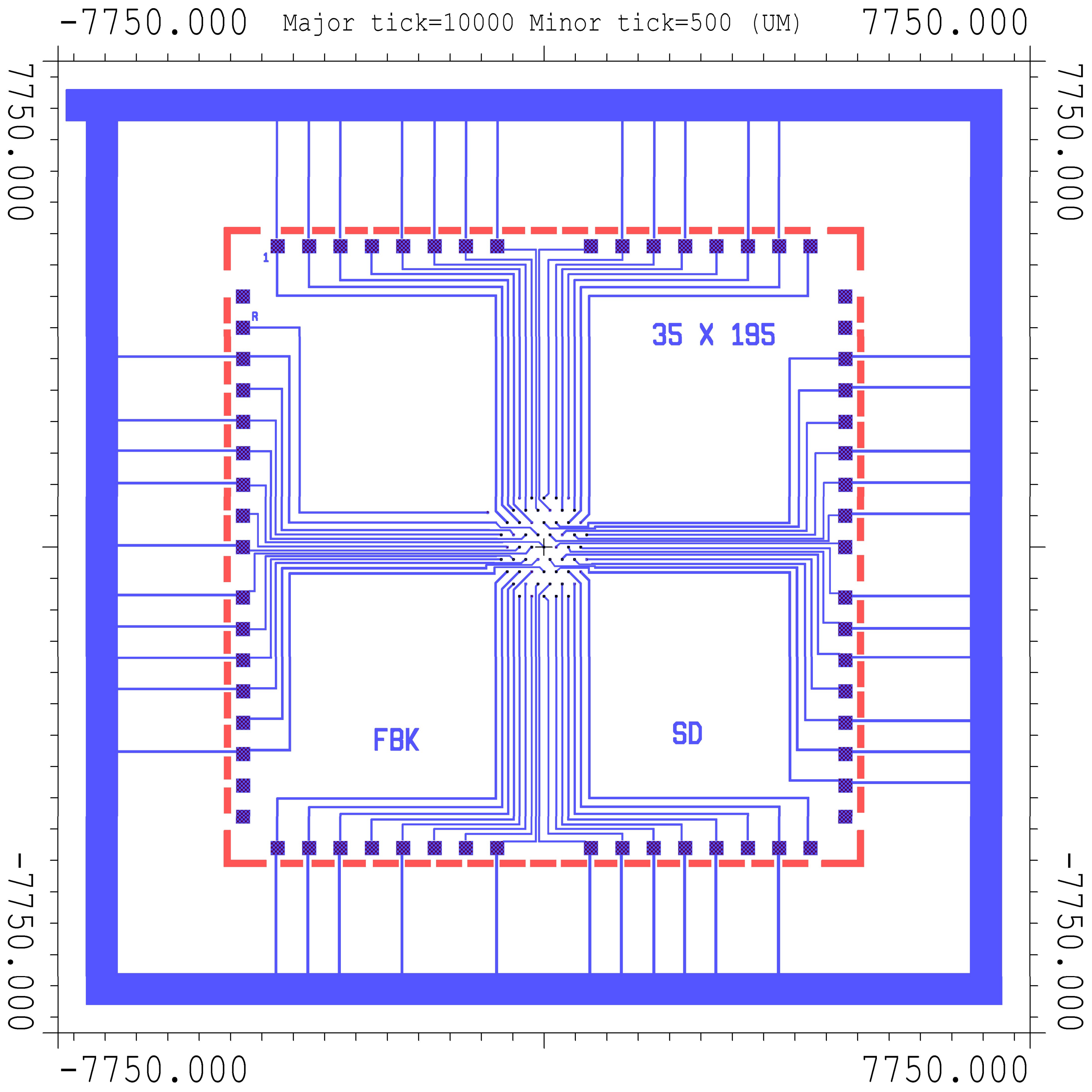

Supplement: Supplementary file 1 [file sensors-24-01251-s001.zip › Layout_Substrate_S3.pdf]
